# Placental ferroptosis and impaired fetal growth in symptomatic and asymptomatic SARS-CoV-2 infections

**DOI:** 10.1042/CS20257407

**Published:** 2026-04-17

**Authors:** Yusmaris Cariaco, Abolfazl Nik-Akhtar, Marie-Eve Brien, Keir Menzies, Sylvie Girard, Shannon Bainbridge

**Affiliations:** 1 Interdisciplinary School of Health Sciences, Faculty of Health Sciences, University of Ottawa, Ottawa, Canada; 2 Ste-Justine Hospital Research Center, Montreal, Quebec, Canada; 3 Department of Obstetrics & Gynecology, Department of Immunology, Mayo Clinic, Rochester, Minnesota, U.S.A.

**Keywords:** COVID-19, Ferritinophagy, Ferroptosis, Iron metabolism, Placenta, SARS-CoV-2

## Abstract

SARS-CoV-2 infection disrupts iron homeostasis in organs such as the lung. Emerging evidence suggests similar dysregulation in the placenta may impair function and fetal growth. Ferroptosis, an iron-dependent form of cell death, is driven by the accumulation of labile ferrous iron, catalyzing the peroxidation of polyunsaturated fatty acids in the absence of sufficient iron storage or antioxidant defenses. This pathway has been implicated in SARS-CoV-2-related tissue damage in other organs and may contribute to placental dysfunction during pregnancy. However, the influence of infection timing, symptom severity, and fetal sex on placental iron regulation remains unexamined. We analyzed placental samples from a cohort of SARS-CoV-2-exposed pregnancies in the first (*n* = 6), second (*n* = 14), or third trimester (*n* = 30), classified by symptom severity (asymptomatic *n* = 23, symptomatic *n* = 27) and fetal sex, alongside unexposed controls (*n* = 47). We assessed placental iron deposition, lipid peroxidation, and mRNA and protein expression of ferroptosis markers. Publicly available RNA-sequencing datasets from human placenta tissue, exposed or not to SARS-CoV-2, were analyzed to assess ferroptosis-related gene expression. SARS-CoV-2 infection, particularly in symptomatic cases, was associated with reduced placental weight and birth weight. Asymptomatic cases showed increased placental iron deposition, which correlated with lower birthweight and was accompanied by elevated expression of nuclear receptor coactivator 4, a cytosolic adaptor protein that mediates the selective autophagic degradation of ferritin (ferritinophagy) and iron release. Placentas from symptomatic patients exhibited evidence of altered iron transport and sex-specific down-regulation of antioxidant defenses. Transcriptomic analyses further suggested widespread disruption of ferroptosis pathways in placentas from infected patients. Our findings reveal that SARS-CoV-2 infection alters placental iron homeostasis and is associated with ferroptosis-related changes, with distinct molecular responses based on timing of infection, symptom severity, and fetal sex.

## Introduction

The coronavirus disease 2019 (COVID-19) pandemic raises significant health concerns, particularly for vulnerable populations such as pregnant individuals. Expectant mothers face a heightened risk of contracting severe acute respiratory syndrome coronavirus 2 (SARS-CoV-2) compared with their non-pregnant counterparts [1]. Moreover, they exhibit an increased likelihood of requiring hospitalization and intensive care unit admission due to COVID-19, a stark contrast with the general population [2]. Furthermore, studies have highlighted that SARS-CoV-2 infection during pregnancy is associated with an elevated risk of adverse outcomes, including preeclampsia, preterm birth, and low birth weight [3]. These risks appear to be influenced by the timing of infection and symptom severity [4]. While most infected pregnant individuals remain asymptomatic [5,6] or present with mild symptoms [7], those who develop symptoms are more likely to require hospitalization, ICU admission, and mechanical ventilation [6,8]. Symptomatic cases also tend to have longer hospital stays [9] and higher rates of non-delivery hospitalizations [10]. Additionally, neonates born to mothers with a history of SARS-CoV-2 infection, or who were symptomatic, during their 3rd trimester frequently exhibit lower birth weight than those born to non-infected mothers [11], driven in large part by infection-mediated placental dysfunction. Despite extensive research, the precise mechanisms through which SARS-CoV-2 infection contributes to placental dysfunction remain largely undefined.

Ferroptosis is a form of programmed cell death driven by iron overload and oxidative stress, characterized by the peroxidation of lipids containing polyunsaturated fatty acids [12] that has been implicated in COVID-19-related tissue damage, and may represent a candidate pathway contributing to placental dysfunction in SARS-CoV-2-infected pregnant individuals. In other organ systems, particularly the lungs, ferroptosis contributes to severe COVID-19 pathology by promoting oxidative stress, lipid peroxidation, and cell death, which exacerbates inflammation and tissue injury [13]. Elevated ferritin levels, a key iron storage protein and inflammatory marker, are consistently observed in COVID-19 patients, with higher levels correlating with disease severity and poor clinical outcomes [14–16]. Additionally, a previous study demonstrated that infection of Vero cells with SARS-CoV-2 for 48 h leads to reduced expression of glutathione peroxidase 4 (GPX4)—a key regulator that prevents ferroptosis—thereby increasing the cells’ susceptibility to this form of programmed cell death [17]. Therapeutic interventions targeting ferroptosis, such as vitamin K analogs that mitigate lipid hydroperoxide accumulation, have been proposed to alleviate COVID-19-related complications [18], underscoring the clinical relevance of this pathway. Given that the placenta is highly metabolically active and dependent on iron homeostasis for proper function, disruptions in iron regulation and oxidative stress could predispose placental cells to ferroptosis, potentially compromising placental integrity and fetal health, as has been implicated in other inflammatory and infectious pregnancy conditions such as preeclampsia and malaria [19–22].

Notably, emerging data suggests a potential role for ferroptosis in the establishment of placental dysfunction following SARS-CoV-2 infection. Increased expression of acyl-CoA synthetase long-chain family member 4 (ACSL4), a key ferroptotic marker, has been observed in SARS-CoV-2-exposed placentas, particularly in regions with detectable viral RNA [23,24]. Additionally, SARS-CoV-2-exposed placentas exhibit evidence of dysregulated iron metabolism, including elevated ferritin levels, up-regulation of iron transport proteins (DMT1, ferroportin), and increased oxidative stress, all of which create a pro-ferroptotic environment [25]. While these initial investigations suggest an association of ferroptosis with COVID-19-associated placental dysfunction, the extent to which this process is linked with adverse pregnancy outcomes remains unclear. Moreover, whether ferroptotic activation varies by maternal symptom status, timing of infection, or fetal sex has yet to be explored.

To address these gaps, the present study investigates placental iron accumulation, lipid peroxidation, and expression of ferroptotic markers in a cohort of patients with prenatal SARS-CoV-2 infection who were symptomatic or asymptomatic, stratified by fetal sex, with associations to pregnancy outcomes. Additionally, a bioinformatic analysis of publicly available RNA sequencing data from human placental tissue, either infected with SARS-CoV-2 *ex vivo* or obtained from patients infected during pregnancy, was performed to examine the expression of ferroptosis-related genes (FRGs).

## Methods

### Patient recruitment and sample collection

Placental specimens were collected at the time of delivery from pregnant patients delivering at Centre Hospitalier Universitaire (CHU) Sainte Justine-CHUSJ in Montreal, QC, Canada, between April 2020 and July 2021. Patients were classified according to SARS-CoV-2 exposure status. SARS-CoV-2 positive individuals included those with a SARS-CoV-2+ PCR test on a nasopharyngeal swab at any point during pregnancy, including at the time of delivery. SARS-CoV-2 negative individuals had no record of a positive SARS-CoV-2 test at any time during pregnancy. For clarity, they are referred to as SARS-CoV-2+ or SARS-CoV-2- throughout the present study. SARS-CoV-2- patients were randomly selected from deliveries occurring within the same timeframe. Universal trimester-based SARS-CoV-2 screening was not performed; therefore, infection status, symptom classification, and timing were determined based on documented positive PCR testing recorded in the medical chart, including routine testing at hospital presentation and at the time of delivery. Asymptomatic infections were defined by a positive PCR test in the absence of documented COVID-19-related symptoms. Historical infection status, including the presence, the classification as symptomatic or asymptomatic, and timing of any previous SARS-CoV-2 positive test during pregnancy, was obtained through medical chart review. Infection timing was therefore inferred from the date(s) of documented positive PCR test(s) and, in the absence of universal trimester-based screening, may not reflect the exact onset of infection—particularly in asymptomatic individuals. A total of 97 placenta tissue biopsies were included in the analysis, consisting of 47 SARS-CoV-2− patients and 50 from SARS-CoV-2 positive patients (*n* = 6, 1st trimester; *n* = 14, 2nd trimester; *n* = 30, 3rd trimester; *n* = 23 asymptomatic and* n* = 27 symptomatic), with accompanying de-identified clinical characteristics datasets. Individualized birthweight centiles were calculated using previously described methods [26], while placental weight centiles were calculated with the Fetal and Placental Biometry Calculator [27] (https://autopsypathology.net/wp-content/uploads/2015/10/1.FetalPlacentalBiometryCalculator_Connolly.html). Placenta samples were fixed in formalin and processed to obtain 5 μm thick sections, flash-frozen for protein/biochemical analyses, or preserved in RNAlater for mRNA measurement.

### Immunohistochemical assessment of placental iron deposition and oxidative damage

Paraffin-embedded placental sections were deparaffinized, rehydrated, and processed for iron staining (Perl’s Prussian blue) or immunohistochemistry (IHC) for ferritin and 4-hydroxynonenal (4-HNE). For iron detection, sections were incubated in a hydrochloric acid-potassium ferrocyanide solution for 30 min, counterstained with nuclear fast red (Abcam, ab246831), washed in distilled water, dehydrated in graded ethanol solutions, and mounted with synthetic resin. IHC for ferritin and 4-HNE was performed using the Leica Bond™ system, following a modified version of the Bond Polymer Refine IHC protocol. Antigen retrieval was performed in sodium citrate buffer (pH 6, Epitope Retrieval Solution 1) for 20 min. Sections were then incubated with rabbit polyclonal anti-ferritin (1:1500 dilution, Invitrogen, PA5-120011) or anti-4-HNE (1:1000 dilution, R&D Systems, MAB3249) antibodies for 30 min at room temperature. Detection was achieved with an HRP-conjugated compact polymer system, followed by DAB chromogen staining, hematoxylin counterstaining, and cover-slipping. All slides were scanned at the Louise Pelletier Histology Core Facility using a Zeiss Axio Scan.Z1 Slide Scanner (20× magnification). For iron accumulation assessments, QuPath [28] v0.5.0 was used to perform pixel-based classification of iron-positive areas, normalized to total tissue area. IHC assessments of ferritin and 4-HNE were performed using the intensity feature plugin in QuPath on villous tissue areas, excluding the intervillous space, with results expressed as mean intensity values.

### Placental expression of ferroptosis-related proteins

Flash-frozen placenta tissue was pulverized, weighed, and lysed in radioimmunoprecipitation (RIPA) buffer supplemented with protease and phosphatase inhibitors (Roche Complete Protease Inhibitor Cocktail #11697498001 and Roche PHOStop Phosphatase Inhibitor Cocktail #4906845001). The tissue was homogenized, adding 10 μl of buffer per mg of tissue, with a Bead Mill 24 Homogenizer (2.8 mm ceramic beads) for three 40-s cycles at 2.09 m/s, with 22-s rest intervals. Lysates were centrifuged (16,000 rcf for 10 min, 4°C), and protein quantification in supernatants using the DC protein assay (Bio-Rad Laboratories) normalized protein concentrations with RIPA buffer and 4× Laemmli buffer, with 10% β-mercaptoethanol. Samples were heated at 95°C for 5 min, and proteins were separated using 8%, 10%, or 12% SDS–PAGE gels (based on molecular weight; TGX Stain-Free Fast Cast Acrylamide Kit). Gels were activated using the Stain-Free setting on the ChemiDoc Imaging System, with proteins transferred onto 0.2 μm nitrocellulose membranes (Bio-Rad) via the Trans-Blot Turbo system (Bio-Rad). Total protein loading was visualized using the stain-free blot setting on the ChemiDoc system. Membranes were then blocked for 1 h at room temperature in 5% BSA in TBS-T buffer and incubated at 4°C overnight with primary antibodies: anti-GPX4, -FTH1, -NCOA4, and -CD98 (rabbit, Cell Signaling #52455, #4393, #66849, and #47213, respectively) and -SLC40A1/ferroportin (mouse, Abcam #ab239583). After washing (3 × 5 min, TBS-T), membranes were incubated for 1 h at room temperature with anti-rabbit (Cell Signaling, #7074) or anti-mouse (Bio-rad #1706516) HRP-conjugated secondary antibodies. Protein bands were detected using Clarity/Clarity Max ECL substrates (Bio-Rad) and imaged on the ChemiDoc system. ImageLab 6.1 software was used for quantification, normalizing protein band intensity to total protein intensity.

### Placental expression of ferroptosis-related genes

RNA was extracted using the TRIzol reagent and quantified with a NanoDrop 2000 spectrophotometer. Subsequently, cDNA was synthesized using SuperScript II reverse transcriptase following the manufacturer’s protocol. qPCR was performed in duplicate using SsoAdvanced Universal SYBR Green Supermix (Bio-Rad). *SLC11A2, PLA2G6, AIFM2, ferritin heavy chain 1 (FTH1), GPX4, and NCOA4* gene expression was quantified and normalized to *ACTB* using the 2^−ΔΔCt^ method. The primer sequences used are listed in Supplementary Table S1.

### Bioinformatic analysis of placental expression of ferroptosis-related genes

SARS-CoV-2-associated changes in FRG expression were further evaluated using four publicly available RNA sequencing (RNA-seq) datasets obtained from GEO database: (1) GSE181238—Bulk RNA sequencing of healthy term placental tissue, processed into cell clusters and infected *ex vivo* with SARS-CoV-2 (MOI:1) for 24 h (*n* = 3 infected; *n* = 3 mock-infected samples) [29]; (2) GSE171995—Bulk RNAseq of placental samples from patients with confirmed SARS-CoV-2 infections at delivery or within one month prior (*n* = 5 SARS-CoV-2+; *n* = 3 control samples) [30]; (3) GSE171381—Single-cell RNAseq of placental tissue from SARS-CoV-2+ (*n* = 2 decidua; *n* = 2 villi) and control patients (*n* = 2 decidua; *n* = 3 villi) [30]; and (4) GSE233557 is a bulk RNA-sequencing dataset derived from samples collected from the fetal compartment of placentas obtained at delivery, all of whom tested positive for SARS-CoV-2 within the preceding three months. The dataset includes seven uninfected controls, three asymptomatic, and three symptomatic SARS-CoV-2+ cases [31]. DESeq2 results, raw or normalized counts, were retrieved from the GEO database for downstream analysis. Raw counts were processed using the DESeq2 package in RStudio (version 2024.12.0+467) for differential expression analysis, where applicable. A curated list of 430 FRGs from the Ferroptosis Database (FerrDb; http://www.zhounan.org/ferrdb) was compared with differentially expressed genes (DEGs) using the list comparison tool (https://molbiotools.com/listcompare.php) to identify overlaps, visualized as Venn diagrams. Volcano plots (EnhancedVolcano package) and heatmaps (pheatmap package) were generated in RStudio. Gene set enrichment analysis (GSEA) was performed via WebGestalt (https://www.webgestalt.org/) using the WikiPathways database for functional pathway analysis. Single-cell RNA sequencing data (GSE171381) were processed using Seurat with raw counts and metadata [30]. Quality control filtering removed low-quality cells/genes, followed by normalization, identification of highly variable features, and principal component analysis for dimensionality reduction. UMAP was used for visualization, and clustering was performed using FindNeighbors and FindClusters. Cluster identities were assigned based on metadata. Differential expression analysis (Wilcoxon rank-sum test, FindMarkers) was applied between SARS-CoV-2+ and control conditions, with FDR correction for multiple comparisons. Violin plots (VlnPlot) and dot plots (ggplot2) were used for visualization.

### Statistical analysis

Differences between two groups were assessed using an unpaired, two-tailed Student’s *t*-test or Mann–Whitney U test for continuous variables and Fisher’s exact test for categorical variables. Normality of continuous variables was evaluated using the Shapiro–Wilk test. For comparisons among multiple groups, one-way analysis of variance (ANOVA) followed by Dunnett’s post-hoc test was used. Results are expressed as mean ± standard error of the mean (SEM). Spearman correlation analyses and Cohen’s *d* effect size calculations were also conducted. A *P*-value <0.05 was considered statistically significant. Data were analyzed using GraphPad Prism 10.

## Results

### Reduced birthweight and placental weight associated with SARS-CoV-2 infection

The present study included 97 pregnant individuals divided into three groups: SARS-CoV-2−, SARS-CoV-2+ asymptomatic (+Asx), and SARS-CoV-2+ symptomatic (+Sx). Demographic data and clinical variables—including maternal age, race, pre-pregnancy body mass index (BMI), medical history, and obstetrical outcomes—were comparable across groups (Table 1). Notably, most infections were detected in the third trimester, with similar timing and distribution in both SARS-CoV-2+ groups (+Asx and +Sx). This demographic and clinical similarity reduces potential confounding and supports interpretation of outcome differences in relation to infection and symptom status.

**Table 1:** Maternal characteristics and obstetrical information of the studied population

Maternal characteristics and obstetrical informations	SARS-CoV-2- (*n* = 47)	SARS-CoV-2 + Asymptomatic (*n* = 23)	SARS-CoV-2 + Symptomatic (*n* = 27)
Maternal age (years)	31 (20-38)	31 (22-45)	33 (24-42)
Race/Ethnicity (%)			
Black	11 (23.4)	9 (39.1)	3 (11.1)
Caucasian	24 (51.1)	6 (26.1)	12 (44.4)
Other	12 (25.5)	8 (34.8)	12 (44.4)
BMI (pre-pregnancy)	25.5 (17.5–36.3)	28.2 (18.6-43.6)	27.2 (17.3-42.3)
Gestational age (weeks)	39.4 (35.7–41.3)	38.5 (32-41)	38.4 (26-41)
Birth weight (g)	3538 (2760–4410)	3188 (1340–4410) *	3128 (890–4050) *
Female	3418 (2760–4040)	3270 (2270–3730)	3165 (2180–3650)
Male	3614 (2830–4410)	3314 (2090–4490)	3103 (890–4050) *
Individualized birth weight centile	57.5 (4.5–98.9)	42.2 (0.6–96.6) *	39.9 (0.1–90.8) **
Female	60.7 (11.0–97.5)	38.8 (0.6–91.5) *	38.2 (0.1–77.9) *
Male	55.7 (4.5–98.9)	45.6 (3.7–96.6)	41.1 (0.8–90.8)
Placental weight (g)	489 (300–681)	464 (299–678)	414 (185-550) ***
Female	476 (357–675)	476 (320–639)	408 (255–455) *
Male	491 (300–681)	466 (312–678)	418 (185–550) *
Placenta weight centile	63 (1–99)	58 (1–99)	41 (1–99)**
Female	65 (2–99)	62 (1–99)	33 (1–67) *
Male	62 (1–99)	55 (1–99)	46 (2–99)
CS delivery (%)	13 (27.7)	11 (47.8)	7 (25.9)
Gestational diabetes (%)	3 (6.4)	6 (26.1)	7 (26.0)
Gestational or chronic hypertension (%)	1 (2.1)	3 (13.0)	0 (0)
Exposure trimester (weeks)	NA	30.7 (4–41)	27.5 (10–40)
Frequency of infection during first trimester (%)	NA	2/23 (8.7)	4/27 (14.8)
Frequency of infection during second trimester (%)	NA	6/23 (26.1)	8/27 (29.6)
Frequency of infection during third trimester (%)	NA	15/23 (65.2)	15/27 (55.6)

Data are presented as mean (range) or *n* (%). BMI: body mass index; GA: gestational age; CS: C-section; SARS-CoV-2: severe acute respiratory syndrome coronavirus 2. Statistical comparisons were made against SARS-CoV-2 negative controls using *t*-tests for normally distributed continuous variables or the Mann–Whitney U test when the Shapiro–Wilk test indicated non-normality. Categorical variables were analyzed using the Chi-square test. Statistical significance is denoted as ^*^P <0.05, ^**^P <0.01, ^***^P <0.001.

Newborns of SARS-CoV-2+ mothers had lower birthweights compared with those born to SARS-CoV-2− mothers. This reduction reached statistical significance in both +Asx and +Sx cases. When stratified by newborn sex, male newborns of +Sx mothers had significantly lower raw birthweights compared with SARS-CoV-2-negative controls, while birthweights among females did not differ significantly from controls. After adjustment using individualized birthweight centiles, centiles were significantly lower in both SARS-CoV-2+ groups overall. Among females, birthweight centiles were significantly reduced in both +Asx and +Sx pregnancies. In contrast, male birthweight centiles did not differ significantly between groups (Table 1). Placental weight was significantly lower only in the +Sx group, and this reduction was observed in both male and female newborns. Placental weight centiles showed a similar pattern, with a significant reduction in the overall +Sx group and among females. No significant differences in placental weight centiles were detected among males.

Together, these findings indicate that maternal SARS-CoV-2 infection, regardless of symptom status, was associated with lower birthweight centiles overall, whereas reductions in placental weight were observed only in symptomatic infection. Sex-specific differences were most evident in females after centile adjustment.

### SARS-CoV-2 infection during pregnancy is associated with placental iron deposition and lipid peroxidation

**Figure 1:**
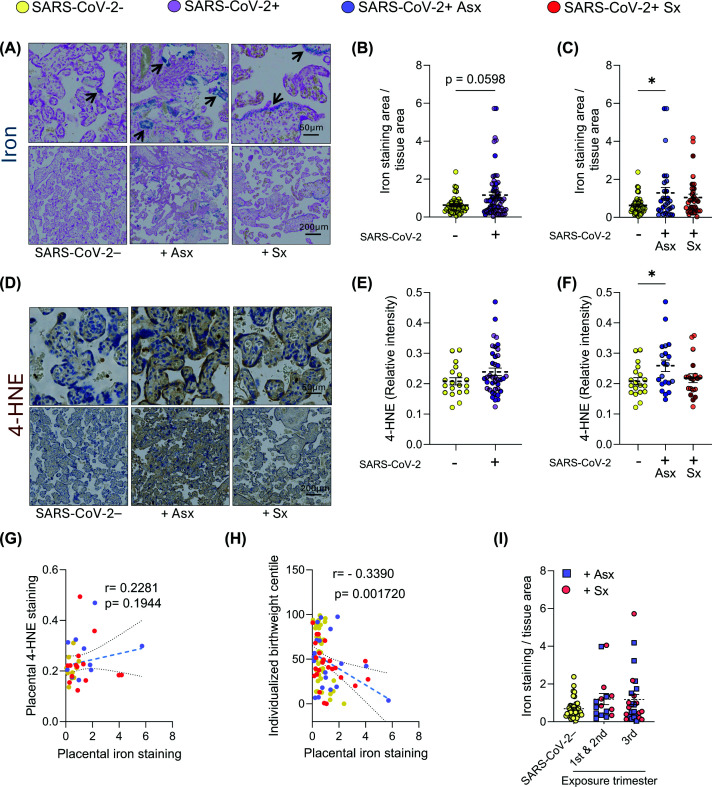
Placentas from asymptomatic cases of prenatal SARS-CoV-2 infection show increased iron deposition and higher lipid peroxidation (**A**) Representative images of iron staining in placental tissues from patients who were SARS-CoV-2− or SARS-CoV-2+ (the latter later stratified into Asx or Sx; abbreviations: −, +Asx, +Sx) during pregnancy. Arrows indicate regions with notable iron deposits. (**B**) Quantification of overall placental iron staining in SARS-CoV-2− versus SARS-CoV-2+ cases. (**C**) Comparison of iron staining across SARS-CoV-2−, +Asx, and +Sx groups. (**D**) Representative images of overall 4-HNE staining in placental tissues. Dark brown staining highlights areas of lipid peroxidation. (**E**) Quantification of placental 4-HNE staining intensity in SARS-CoV-2− and SARS-CoV-2+ cases. (**F**) Comparison of 4-HNE staining intensity in SARS-CoV-2−, +Asx, and +Sx groups. (**G**) Spearman correlation test between placental 4-HNE and iron staining in SARS-CoV-2− (yellow), +Asx (blue), and +Sx (red) cases. (**H**) Spearman correlation test between birthweight centiles and placental iron staining in SARS-CoV-2− (yellow), +Asx (blue), and +Sx (red) cases. (**I**) Placental iron accumulation based on exposure trimester. Darker dots (B,C,E,F) indicate samples from patients who tested positive for SARS-CoV-2 at delivery. Statistical analysis was performed using the Mann–Whitney test (B,E) or one-way ANOVA followed by Dunnett’s multiple comparisons test (C,F). *: *P* <0.05, indicating statistical significance.

Compared with SARS-CoV-2− controls, placentas from SARS-CoV-2+ cases showed a notable trend toward elevated iron accumulation with a moderate effect size (Cohen’s *d* = 0.52) (Figure 1A,B) and no significant changes in 4-HNE levels (Figure 1D,E), a marker of lipid peroxidation. Stratifying by symptom status revealed increased iron deposition and 4-HNE levels in +Asx cases, whereas the +Sx group showed a similar trend that did not reach significance (Figure 1C,F). Darker data points in Figure 1B–F represent individuals who tested SARS-CoV-2 positive at delivery. These cases did not differ noticeably from those with earlier infections, as both recent and earlier infections were evenly distributed across the full range of observed values. Placental iron levels showed a weak positive correlation with 4-HNE levels (Figure 1G) and a statistically significant negative correlation with individualized birthweight centile (Figure 1H) in the overall dataset (Figure 1G,H). When these correlations were examined separately for SARS-CoV-2−, +Asx, and +Sx groups, the associations were no longer statistically significant, likely due to reduced sample size, but the direction of the relationships was preserved across all groups (Supplementary Figure S1).

To further explore whether the timing of SARS-CoV-2 infection influenced placental iron accumulation, individuals infected during the first and second trimesters were grouped together, due to smaller numbers, and compared with those infected during the third trimester as well as to SARS-CoV-2− controls. With +Asx and +Sx cases distributed evenly across all data points, the timing of infection did not substantially affect placenta iron content (Figure 1I). Rather, the presence of infection—particularly when considering symptom status—seemed to be more closely associated with placental iron dysregulation.

### Ferritinophagy is associated with iron dysregulation and ferroptosis in placentas from pregnancies affected by prenatal SARS-CoV-2 infection

To elucidate mechanisms driving iron accumulation, we assessed expression of ferritin (FTH1, iron storage protein) and nuclear receptor coactivator 4 (NCOA4, ferritinophagy executer), two key regulators of iron homeostasis. Ferritin plays a crucial role in maintaining intracellular iron balance, while NCOA4 mediates the selective autophagic degradation of ferritin, leading to the release of free iron.

**Figure 2:**
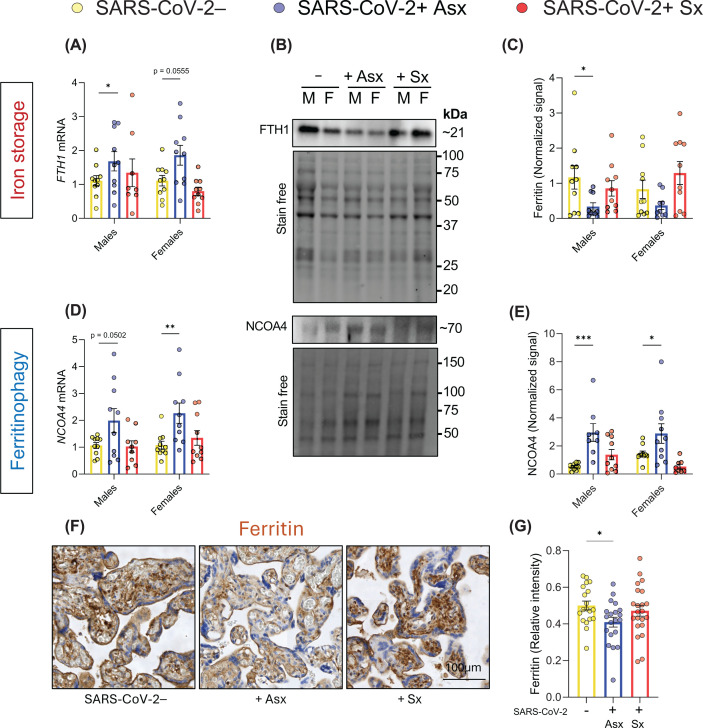
Ferritinophagy as a potential mechanism driving placental ferroptosis in asymptomatic SARS-CoV-2 infection Relative mRNA expression (**A,D**) and protein quantification (**C,E**) of FTH1 and NCOA4 in placentas from SARS-CoV-2− (−), asymptomatic SARS-CoV-2+ (+Asx), and symptomatic SARS-CoV-2+ (+Sx) groups. (**B**) Representative bands from western blot analysis in placental tissues. M: males, F: females. (**F,G**) Representative images and quantification of overall FTH1 staining intensity in placenta histological sections. Bar graphs display individual data points and mean ± SEM. Statistical analysis was performed using one- (F) or two-way ANOVA (A–E) followed by Dunnett’s multiple comparisons test. *: *P* <0.05, **: *P* <0.01, ***: *P* <0.001.

*FTH1* mRNA expression was significantly up-regulated in placentas from +Asx individuals, particularly in placentas from male newborns (Figure 2A). Ferritin protein levels were significantly decreased in male placentas from +Asx cases (Figure 2B,C), suggesting post-translational depletion. Consistent with this, NCOA4 expression was elevated at both the mRNA and protein levels in placentas of both sexes in +Asx cases (Figure 2B,D,E). Immunostaining confirmed decreased ferritin protein levels in this group (Figure 2F,G), with prominent expression in villous mesenchymal core cells. Together, these findings suggest enhanced NCOA4-mediated ferritinophagy as a driver of iron dysregulation and potential oxidative stress in placentas from asymptomatic SARS-CoV-2+ cases.

### Prenatal SARS-CoV-2 infection alters placental iron transport and ferroptosis-related antioxidant defenses

**Figure 3:**
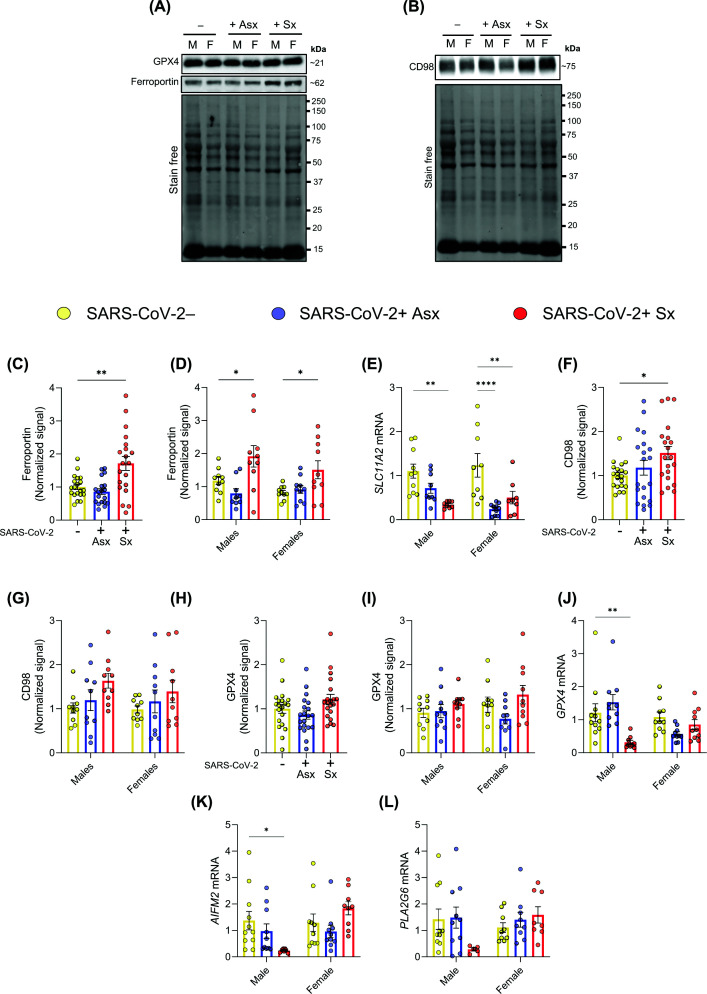
Modulation of ferroptosis in placentas from symptomatic SARS-CoV-2-positive patients (**A,B**) Representative bands from western blot analysis of ferroportin, GPX4, and CD98 in placental tissues from SARS-CoV-2− (−), asymptomatic SARS-CoV-2+ (+Asx), and symptomatic SARS-CoV-2+ (+Sx) groups. M: males, F: females. (**C,D**) Protein quantification of ferroportin. (**E**) Relative mRNA expression of *SLC11A2*. (**F–I**) Protein quantification of CD98 (F,G) and GPX4 (H,I). (**J–L**) Relative mRNA expression of *GPX4*, *AIFM2*, and *PLA2G6.* Bar graphs display individual data points and mean ± SEM. Statistical analysis was performed using one- (for symptomatic stratification) or two-way ANOVA (for symptomatic status and newborn sex stratification) followed by Dunnett’s multiple comparisons test. *: *P* <0.05, **: *P* <0.01, ****: *P* <0.0001.

To help explain distinct placental responses according to symptom status in SARS-CoV-2+ cases, we examined regulators of iron and amino acid transport and antioxidant defense. Ferroportin, the only known iron exporter, protein expression was significantly increased in both female and male placentas from +Sx patients but not in +Asx cases (Figure 3A,C,D), suggesting that iron export may underlie their lower iron burden. In contrast, *SLC11A2* (DMT1) mRNA, a key iron importer, was down-regulated in male placentas from +Sx cases and in female placentas across both SARS-CoV-2+ groups (Figure 3E), indicating impaired iron import.

Amino acid transport plays a key role in oxidative stress resistance. CD98 protein expression, which supports glutathione synthesis via cystine import, was elevated in placentas from +Sx patients, though sex-specific differences were not statistically significant (Figure 3B,F,G). While GPX4 protein levels remained unchanged across groups (Figure 3A,H,I), male placentas from +Sx individuals exhibited significant transcriptional down-regulation of key ferroptosis regulators—*GPX4* and *AIFM2* (Figure 3J,K). *PLA2G6* also showed reduced expression in +Sx cases, though this did not reach statistical significance (Figure 3L)—suggesting reduced antioxidant capacity and increased susceptibility to oxidative stress in the placentas of male offspring.

### Bioinformatic analysis identified ferroptosis dysregulation as a hallmark of SARS-CoV-2-exposed placentas

**Figure 4:**
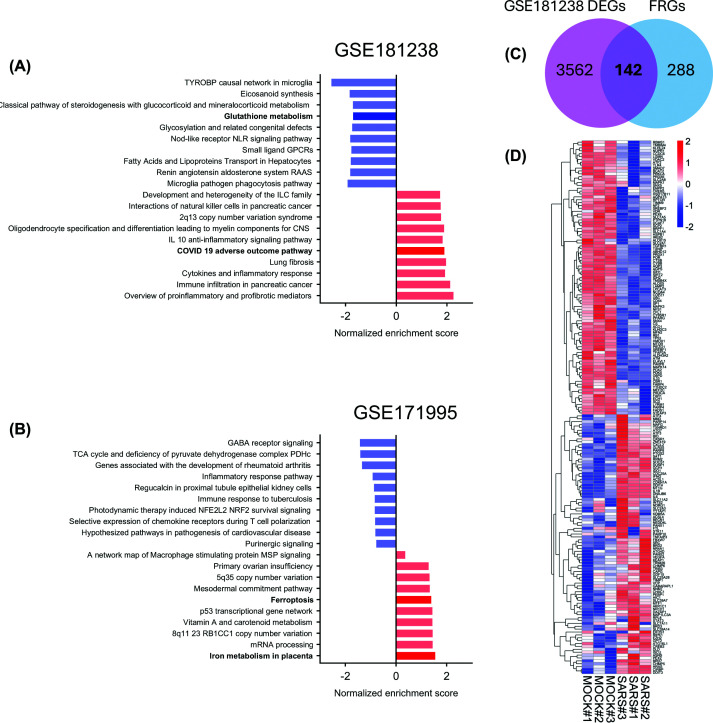
Ferroptosis-related pathways are dysregulated in SARS-CoV-2-infected placental cells and placentas from pregnancies with confirmed infection GSEA of DEGs derived from two independent placental transcriptomic datasets. (**A**) GSE181238 consists of bulk RNA-sequencing data from healthy term placental tissue that was processed into cell clusters and infected *ex vivo* with SARS-CoV-2 (SARS) for 24 h, compared with mock-infected controls (MOCK), and (**B**) GSE171995 consists of bulk RNA-sequencing data from placental samples collected from patients with confirmed SARS-CoV-2 infection at delivery or within a month prior, compared with uninfected controls. In both cases, the top 20 pathways are ranked by normalized enrichment scores, with red bars representing up-regulated pathways and blue bars representing down-regulated pathways. (**C**) Venn diagram showing the overlap between DEGs from GSE181238 and FRGs. A total of 142 genes are shared between the two sets. (**D**) Heatmap of the 142 shared genes from panel (C), displaying the expression profiles across samples. Scale bar represents *z*-scores; red indicates up-regulation, while blue indicates down-regulation.

To contextualize the association between iron dysregulation, placental stress signatures, and fetal growth measures observed in our cohort, we reanalyzed publicly available transcriptomic datasets from *ex vivo* and *in vivo* studies of placental tissue in the context of prenatal SARS-CoV-2 infection. Using the GSE181238 *ex vivo* exposure dataset, gene set enrichment analysis revealed suppression of glutathione metabolism (Figure 4A), of relevance as glutathione plays a critical role in preventing ferroptosis by neutralizing lipid peroxidation and maintaining redox homeostasis. Gene set enrichment of the COVID-19 adverse outcome pathway was also observed. Among 3562 DEGs, 142 overlapped with FRGs, showing up-regulation of cytokines (e.g., IL6 and IL1B) and ferroptosis markers (e.g., PTGS2 and CHAC1) in these experimentally infected samples (Figure 4C,D).

**Figure 5:**
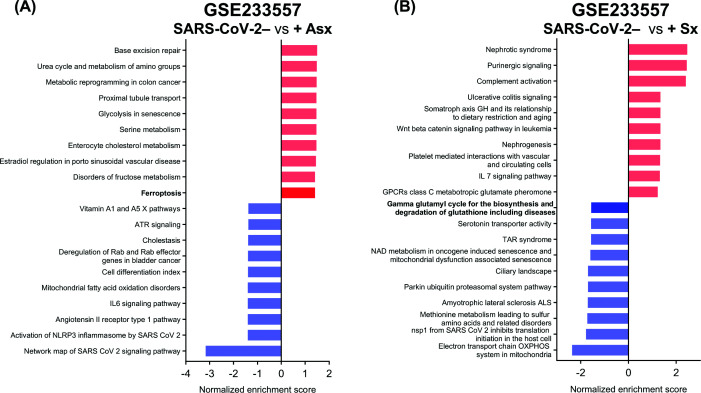
Asymptomatic SARS-CoV-2 infection is associated with enrichment of the placental ferroptosis pathway GSEA of DEGs from the GSE233557 dataset. This dataset comprises bulk RNA-seq profiles from the fetal compartment of placentas, including seven COVID-negative (SARS-CoV-2−), three asymptomatic COVID-positive (SARS-CoV-2+Asx), and three symptomatic SARS-CoV-2+ (SARS-CoV-2+Sx) cases. The top 20 enriched pathways from SARS-CoV-2− versus SARS-CoV-2+Asx (**A**) or SARS-CoV-2− versus SARS-CoV-2+Sx (**B**) are ranked by normalized enrichment score; red bars indicate pathways enriched in samples from SARS-CoV-2+ patients, while blue bars indicate pathways down-regulated relative to controls.

Similarly, analysis of the GSE171995 bulk RNA-seq dataset from *in vivo* SARS-CoV-2-infection samples confirmed enrichment of iron metabolism and ferroptosis pathways in placentas from individuals who tested positive for SARS-CoV-2 at delivery or within the last month of pregnancy (Figure 4B). Furthermore, analysis of the GSE233557 dataset, which includes fetal placenta samples from individuals—most with active SARS-CoV-2 infection at delivery—stratified by symptomatic status, revealed distinct pathway alterations. Ferroptosis emerged as one of the top activated pathways in asymptomatic cases compared with uninfected controls (Figure 5A), while symptomatic infections were associated with down-regulation of the gamma-glutamyl cycle, a pathway involved in glutathione biosynthesis and redox homeostasis (Figure 5B). These findings are consistent with the results of the present study, suggesting that ferroptosis-related pathways are more prominently enriched in asymptomatic infections, whereas impaired glutathione-related antioxidant defense is more pronounced in symptomatic cases. These results suggest ferroptosis dysregulation-driven by inflammatory and metabolic disturbances—may contribute to placental oxidative stress signatures associated with reduced birthweight in SARS-CoV-2-exposed pregnancies, especially in asymptomatic cases.

### Single-cell RNAseq reveals cell-type specific ferroptosis signatures and iron dysregulation in placentas from SARS-CoV-2-infected cases

**Figure 6:**
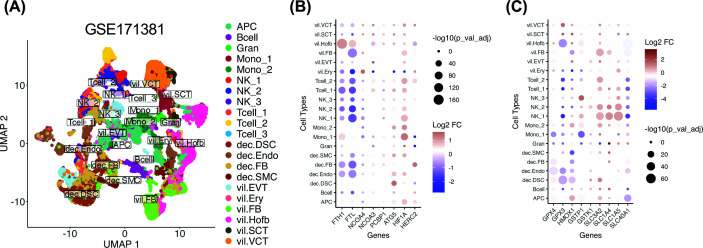
Cell-specific dysregulation of FRGs in placentas from SARS-CoV-2-infected individuals A publicly available single-cell RNA sequencing dataset (GSE171381) was analyzed, representing diverse villous (vil) and decidual (dec) cell types, including maternal and fetal immune cells. The UMAP plot (**A**) illustrates cell clustering across these populations. Differential expression analysis was performed between placenta cells from SARS-CoV-2+ patients (*n* = 2 decidual, *n* = 2 villous samples) and uninfected controls (*n* = 2 decidual, *n* = 3 villous samples). Dot plots (**B,C**) highlight DEGs associated with ferritinophagy, antioxidant response/glutathione metabolism, and iron export. Panel B shows ferritinophagy-related genes, whereas panel C shows antioxidant response/glutathione metabolism and iron export genes. Up-regulated genes are shown in red, down-regulated genes in blue, and dot size represents statistical significance after multiple comparison correction. dec.DSC (decidual stromal cell), dec.Endo (decidual endothelial cell), dec.SMC (decidual smooth muscle cell), dec.FB (decidual fibroblast), vil.FB (villous fibroblast), vil.EVT (villous extravillous trophoblast), vil.VCT (villous cytotrophoblast), vil.SCT (villous syncytiotrophoblast), vil.Ery (villous erythrocyte/blast), vil.Hofb (villous Hofbauer cell), APC (antigen-presenting cell), Mono_1 (monocyte subtype 1), Mono_2 (monocyte subtype 2), Gran (granulocyte), NK_1 (natural killer cell subtype 1), NK_2 (natural killer cell subtype 2), NK_3 (natural killer cell subtype 3), Tcell_1 (T cell subtype 1), Tcell_2 (T cell subtype 2), Tcell_3 (T cell subtype 3), and Bcell (B cell).

To assess ferroptosis-related changes at single-cell resolution, we analyzed scRNA-seq data from placentas of individuals with symptomatic infection and generated a UMAP plot, using the GSE171381 dataset (Figure 6A). *FTL* (ferritin light chain) and *FTH1* (ferritin heavy chain) expression was reduced by 41% and 28%, respectively, in decidual endothelial cells and by 20% and 14%, respectively, in villous cytotrophoblast, but remained high in phagocytes, including monocytes and Hofbauer cells, consistent with their roles in iron sequestration and erythrophagocytosis (Figure 6B and Supplementary Figure S2). Ferritinophagy regulators (*NCOA4*, *NCOA3*, *PCBP1*, *ATG5*, *HIF1A*, and *HERC2*) were consistently up-regulated in villous trophoblasts, where ferritin genes were suppressed (Figure 6B and Supplementary Figure S2), suggesting an active role for ferritinophagy in iron depletion. Antioxidant enzymes, such as *GPX4* and *HMOX1*, were up-regulated in villous cytotrophoblasts and syncytiotrophoblasts but down-regulated in most other cell types, including immune cells. Glutathione pathway genes (*GSTP1*, *GSTK1*) and amino acid transporters (*SLC3A2*, *SLC1A4*, *SLC1A5*) were variably up-regulated in villous trophoblasts (Figure 6C and Supplementary Figure S3). Conversely, ferroportin (*SLC40A1*) was down-regulated across cell types, suggesting intracellular iron accumulation and heightened ferroptosis susceptibility (Figure 6C and Supplementary Figure S3).

## Discussion

The present study provides new insights into how SARS-CoV-2 infection during pregnancy may differentially affect placental iron metabolism, suggesting distinct ferroptosis-related responses in asymptomatic versus symptomatic cases, although infection timing—particularly in asymptomatic individuals—could not be precisely determined in the absence of universal trimester-based screening. We show that asymptomatic infection is associated with increased placental iron accumulation and lipid peroxidation, likely driven by NCOA4-mediated ferritinophagy and reduced ferritin protein levels. In contrast, symptomatic cases demonstrated a divergent profile, marked by increased ferroportin and CD98 protein expression—suggesting a potential compensatory response that enhances iron export and antioxidant defenses and may modulate susceptibility to ferroptosis.

Previously, we demonstrated that SARS-CoV-2 infection induced a unique placental inflammatory signature, characterized by differential expression of IL-8, IL-1β, IFN-γ, IL-1Ra, and CRP according to symptom severity [32]. Cytokines are known to limit the release of cellular iron into circulation, contributing to iron dysregulation. For instance, IFN-γ promotes iron retention in human monocytes through the down-regulation of ferritin, while IL-6 induces hepcidin, leading to ferroportin degradation and iron sequestration [33]. Given the well-established ability of inflammation to disrupt iron homeostasis [34]—exemplified by Parvovirus B19 (B19V)-induced anemia and alterations in iron transport proteins [35]—our present findings suggest that SARS-CoV-2 may exploit similar pathways.

Ferritin is an acute-phase reactant that rises with inflammation. Notably, systemic ferritin is elevated in SARS-CoV-2 infection [14], including in pregnant individuals [36], and correlates with disease severity [37,38], yet our results show reduced ferritin in placentas from asymptomatic cases, highlighting distinct regulation at the placental level. This discrepancy may reflect the enhanced intracellular ferritin degradation via ferritinophagy. Our data support a model in which asymptomatic SARS-CoV-2 infection activates NCOA4-dependent ferritinophagy, releasing iron into the labile pool, where it catalyzes the Fenton reaction and generates hydroxyl radicals, thereby being consistent with increased lipid peroxidation and ferroptosis-related signaling, as indicated by increased 4-HNE levels. These findings align with a prior report of elevated placental iron and lipid peroxidation in third-trimester infections [25], although without distinguishing symptom status—a key novel aspect of our study.

Symptomatic infections, by contrast, had increased placenta ferroportin expression, suggesting enhanced iron efflux [39], potentially as a protective response against iron overload. While this may reduce intracellular iron and mitigate placental ferroptosis, it raises the possibility of increased iron transfer to the fetus—a factor linked to growth restriction [40], developmental abnormalities, and fetal demise [41]. Despite this putative adaptation, our data show a trend toward placental iron overload even in symptomatic cases, which may be associated with lower birthweights observed. Up-regulation of CD98 in placentas from symptomatic cases may help stabilize system xc- to promote cysteine uptake and glutathione synthesis [42], sustaining GPX4 activity and providing partial ferroptosis resistance. However, transcriptional down-regulation of *GPX4*, *AIFM2*, and *PLA2G6* (anti-ferroptotic enzymes [43–45]) in male placentas suggests a fetal sex-specific vulnerability to ferroptotic signals in symptomatic cases, although these sex-stratified analyses were performed within a retrospective cohort with a fixed sample size and may therefore have been underpowered to detect modest effect sizes.

Despite the analyzed scRNA-seq dataset representing symptomatic cases only, gene expression profiles appeared to parallel our findings in asymptomatic placentas, suggesting a shared ferroptotic signature across COVID-19 severities, potentially influenced by cellular context or resolution of detection methods. To further explore transcriptional changes associated with symptomatic status, we analyzed an independent bulk RNA-seq dataset [30], which indicated a ferroptosis signal predominantly in asymptomatic patients, whereas glutathione synthesis pathways were more clearly impaired in symptomatic cases. These results are broadly consistent with our observations. Notably, while that study did not report neonatal birthweights to allow for direct comparison with our findings, it did report that two of three neonates born to asymptomatic individuals developed respiratory distress syndrome—even when maternal infection occurred earlier in pregnancy [30].

Our work supports the possibility that ferroptosis is a central mechanism of tissue damage in SARS-CoV-2 infection, in line with reports in COVID-19-associated lung pathology. In non-pregnant individuals, the expression of ferroptosis markers correlates with lung damage severity, while limited evidence of necrosis, necroptosis, apoptosis, and pyroptosis is observed [13]. Consistent with our findings, previous studies have reported elevated lipid peroxidation in placentas from SARS-CoV-2+, although the relationship to symptom severity remains unclear [46,47]. Previous reports of increased ACSL4 (ferroptosis marker) expression in infected syncytio- and cytotrophoblasts [48] suggest SARS-CoV-2-induced ferroptosis susceptibility, particularly as this increased expression often co-localized to areas of present viral RNA [24]. Additionally, impaired oxidative defense systems—including down-regulated glutathione synthetase, glutathione reductase, mitochondrial function genes [49], and selenocysteine synthesis genes [30]—point to a broader vulnerability of the placenta to ferroptotic stress, particularly in symptomatic cases. These molecular changes are supported by bulk RNA-seq and single-cell RNA-seq bioinformatic analysis of *ex vivo* and *in vivo* SARS-CoV-2 infection, showing patterns consistent with ferritin down-regulation and ferritinophagy gene up-regulation in trophoblasts, along with disrupted selenocysteine and glutathione-related antioxidant pathways. Together, these data suggest a converging pattern of iron and redox imbalance in placentas from SARS-CoV-2-infected individuals across methodologies and patient populations, although causal mechanisms remain to be established.

Given these findings, iron-targeted therapies, including iron chelation, may represent a potential venue for further investigation. Iron chelators have shown benefit in conditions involving viral infection [50] or inflammation, including sepsis, ischemia-reperfusion injury, and systemic inflammatory response syndrome [51], and may inhibit viral replication while limiting iron-induced oxidative damage [52,53]. The lower risk of COVID-19 in individuals with blood type O—who tend to have lower serum iron levels [54,55]—further supports the plausibility of this hypothesis, though experimental validation, particularly in pregnancy models, is needed.

In summary, our study indicates that SARS-CoV-2 infection may disrupt placental iron homeostasis through differential patterns depending on symptom severity. Asymptomatic infections are associated with molecular patterns consistent with enhanced ferritinophagy and ferroptosis-related signaling, while symptomatic infections show indications of alternate pathways involving increased iron export and glutathione maintenance, with fetal sex influencing susceptibility. These findings propose that ferroptosis-related pathways may contribute to COVID-19-related pregnancy complications and identify iron metabolism as a candidate area for further therapeutic exploration aimed at mitigating fetal risk.

## Clinical perspectives

- The present study was undertaken to elucidate how SARS-CoV-2 infection during pregnancy may contribute to placental dysfunction and adverse perinatal outcomes, with a focus on ferroptosis—a form of regulated cell death driven by iron overload and oxidative stress.
- The findings suggest distinct, symptom-specific mechanisms of placental injury: asymptomatic infections were marked by increased placental iron deposition and lipid peroxidation, with molecular patterns consistent with enhanced ferritinophagy and ferroptosis-related signaling, while symptomatic infections exhibited altered iron transport and impaired antioxidant defenses. Both infection types were associated with reduced birth weight, with fetal sex-specific vulnerabilities.
- These results underscore a potential association between maternal SARS-CoV-2 infection and altered placental health and stress pathways. By identifying ferroptosis-related pathways as potential contributors to placental pathology in SARS-CoV-2-exposed pregnancies—especially in asymptomatic cases—the present study highlights iron homeostasis as a candidate pathway for future investigation in pregnancy complications.

## Supplementary Material

Supplementary Figures S1-S3 and Table S1

## Data Availability

RNA-seq datasets analyzed here are publicly available from the NCBI Gene Expression Omnibus (GEO) (https://www.ncbi.nlm.nih.gov/geo) under accession numbers GSE181238(29), GSE171995(30), GSE171381(30) and GSE233557(31). Additional data generated in the present study are available from the corresponding authors upon request.

## Open Access

Open access for this article was enabled by the participation of University of Ottawa in an all-inclusive *Read & Publish* agreement with Portland Press and the Biochemical Society under an agreement with OCUL.

## Abbreviations

ACSL4: acyl-CoA synthetase long-chain family member 4
BMI: body mass index
CHU: Centre Hospitalier Universitaire
COVID-19: coronavirus disease 2019
DEGs: differentially expressed genes
FTH1: ferritin heavy chain 1
FRGs: ferroptosis-related genes
GSEA: Gene set enrichment analysis
IHC: immunohistochemistry
MOI: multiplicity of infection
NCOA4: nuclear receptor coactivator 4
RIPA: radioimmunoprecipitation
RNA-seq: RNA sequencing
SARS-CoV-2: severe acute respiratory syndrome coronavirus 2
+Asx: SARS-CoV-2+ asymptomatic
+Sx: SARS-CoV-2+ symptomatic
scRNA-seq: single-cell RNA sequencing
SEM: standard error of the mean
UMAP: Uniform Manifold Approximation and Projection
