# Supplementary material for: Placental ferroptosis and impaired fetal growth in symptomatic and asymptomatic SARS-CoV-2 infections
Source: Clin Sci (Lond). 2026 Apr 17;140(5):681–97. doi: 10.1042/CS20257407 (PMC13142940; doi:10.1042/CS20257407)
Supplement: Supplementary Figures S1-S3 and Table S1 [file CS-2025-7407_supp.pdf]

Supplementary table 1. List of primers used in this study

Gene	Forward Primer	Reverse Primer
SLC11A2	TGGAGATCATGGGGAGTCTG	AAGAAAACCTGGTCCGGTGAA
PLA2G6	CATCACAGCCGTATCATCAGC	TCGGTGACATCCATCTGAGTG
AIFM2	AGACAGGGTTCGCCAAAAAGA	CAGGTCTATCCCCACTACTAGC
FTH1	CCCCCATTTGTGTGACTTCAT	GCCCGAGGCTTAGCTTTCATT
GPX4	GAGGCAAGACCGAAGTAAACTAC	CCGAACTGGTTACACGGGAA
NCOA4	GCTCAGCAGCTCTACTCGTTA	GGCACACAGAGACTTGATTGG

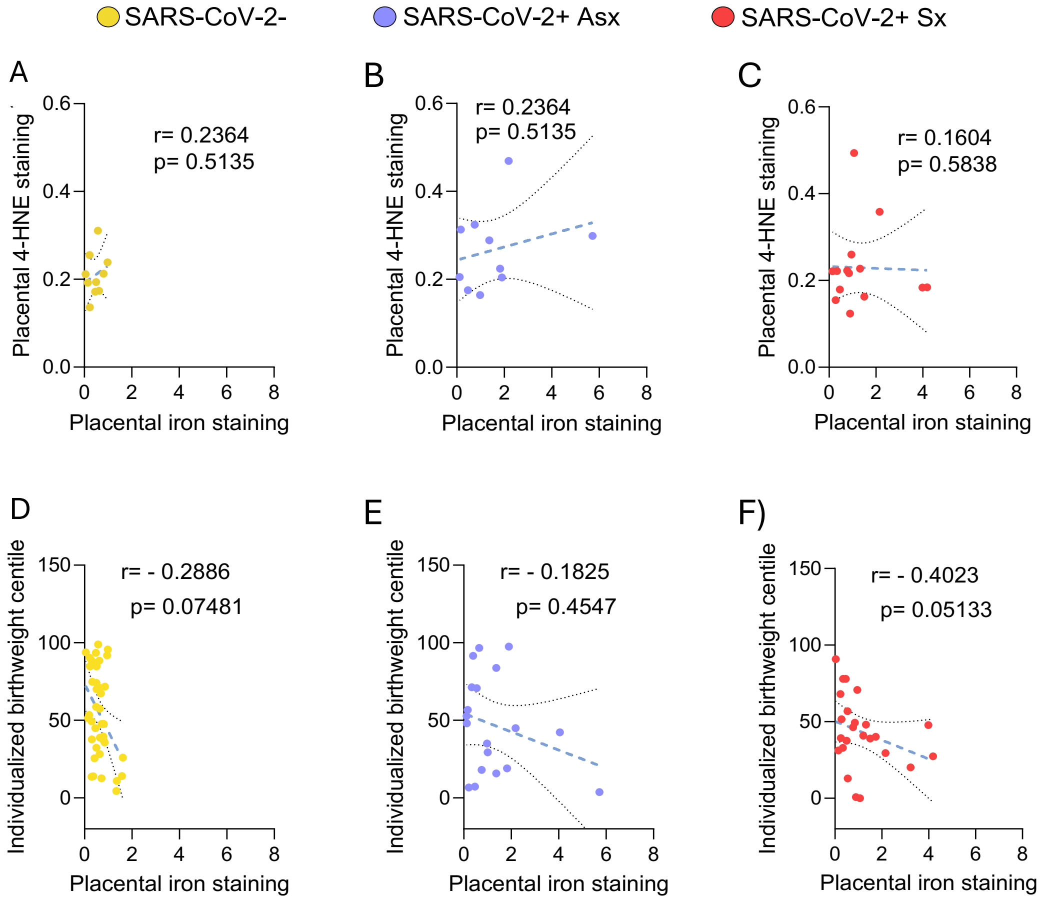

Supplementary Figure 1. Correlation of placental iron staining with oxidative stress (4-HNE) and individualized birthweight centile across SARS-CoV-2−, SARS-CoV-2+ Asx, and SARS-CoV-2+ Sx pregnancies. Scatter plots show correlations between placental iron staining and 4-HNE staining (A–C) and individualized birthweight centile (D–F) in SARS-CoV-2− (yellow), SARS-CoV-2+ asymptomatic (blue), and SARS-CoV-2+ symptomatic (red) pregnancies. Regression lines with 95% confidence intervals are shown, along with Spearman r and p-values. Although subgroup correlations did not reach statistical significance, the direction of associations was consistent with the overall dataset.

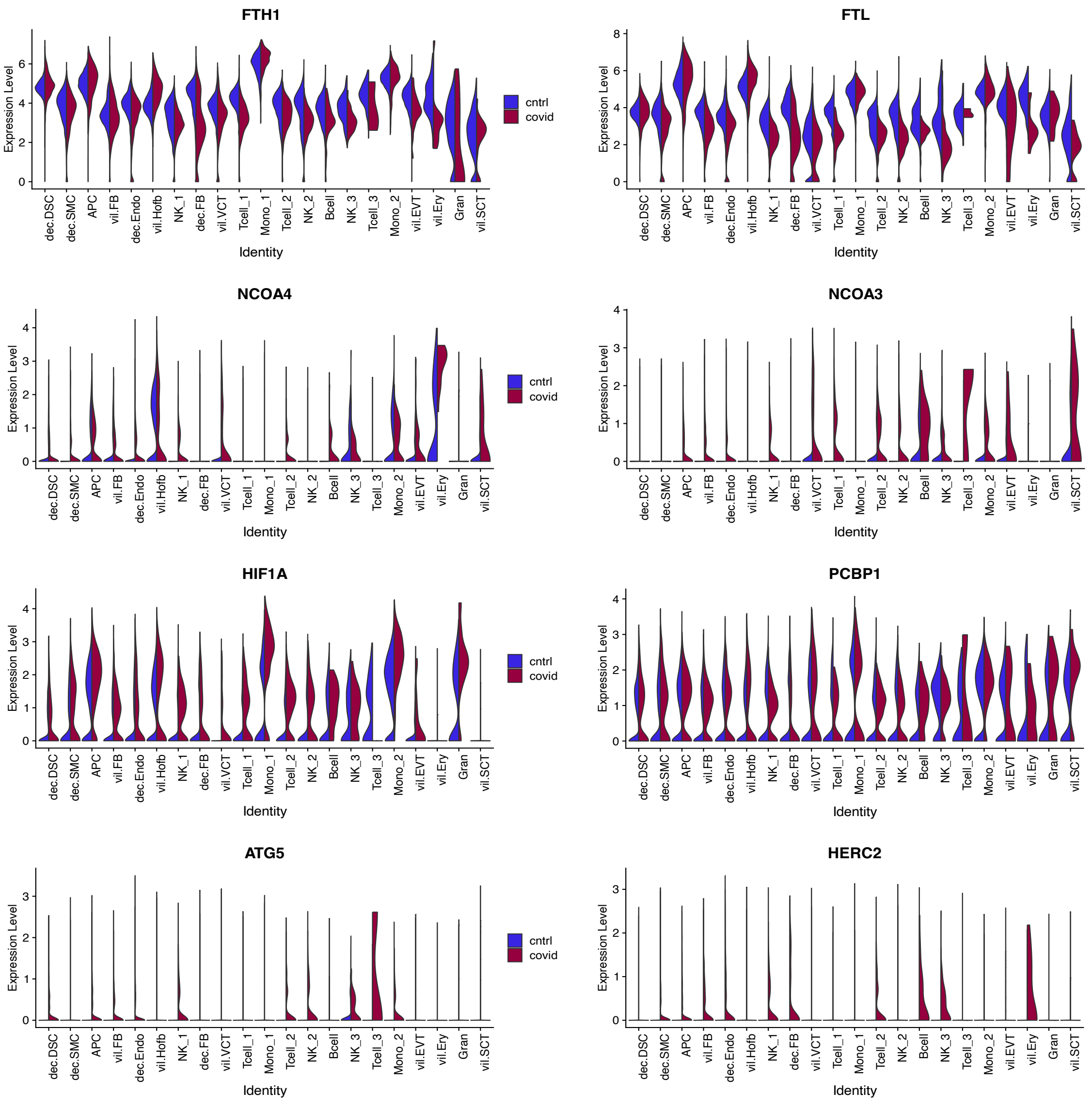

Supplementary Figure 2. Cell-type specific expression profiles of ferritinophagy-related genes in placentas from SARS-CoV-2 infected and uninfected individuals. Violin plots display the mean single-cell RNA expression levels of genes involved in ferritinophagy across distinct placental cell types from SARS-CoV-2–positive (covid, red) and control (cntrl, blue) cases. Data are derived from the GSE171381 dataset and correspond to the cell clusters shown in Figure 6B. Expression patterns of ferritin heavy (FTH1) and light (FTL) chains, NCOA4, NCOA3, HIF1A, PCBP1, ATG5, and HERC2 are shown. Cell populations include dec.DSC (decidual stromal cell), dec.Endo (decidual endothelial cell), dec.SMC (decidual smooth muscle cell), dec.FB (decidual fibroblast), vil.FB (villous fibroblast), vil.EVT (villous extravillous trophoblast), vil.VCT (villous cytotrophoblast), vil.SCT (villous syncytiotrophoblast), vil.Ery (villous erythrocyte/blast), vil.Hofb (villous Hofbauer cell), APC (antigen-presenting cell), Mono_1 (monocyte subtype 1), Mono_2 (monocyte subtype 2), Gran (granulocyte), NK_1 (natural killer cell subtype 1), NK_2 (natural killer cell subtype 2), NK_3 (natural killer cell subtype 3), Tcell_1 (T cell subtype 1), Tcell_2 (T cell subtype 2), Tcell_3 (T cell subtype 3), Bcell (B cell).

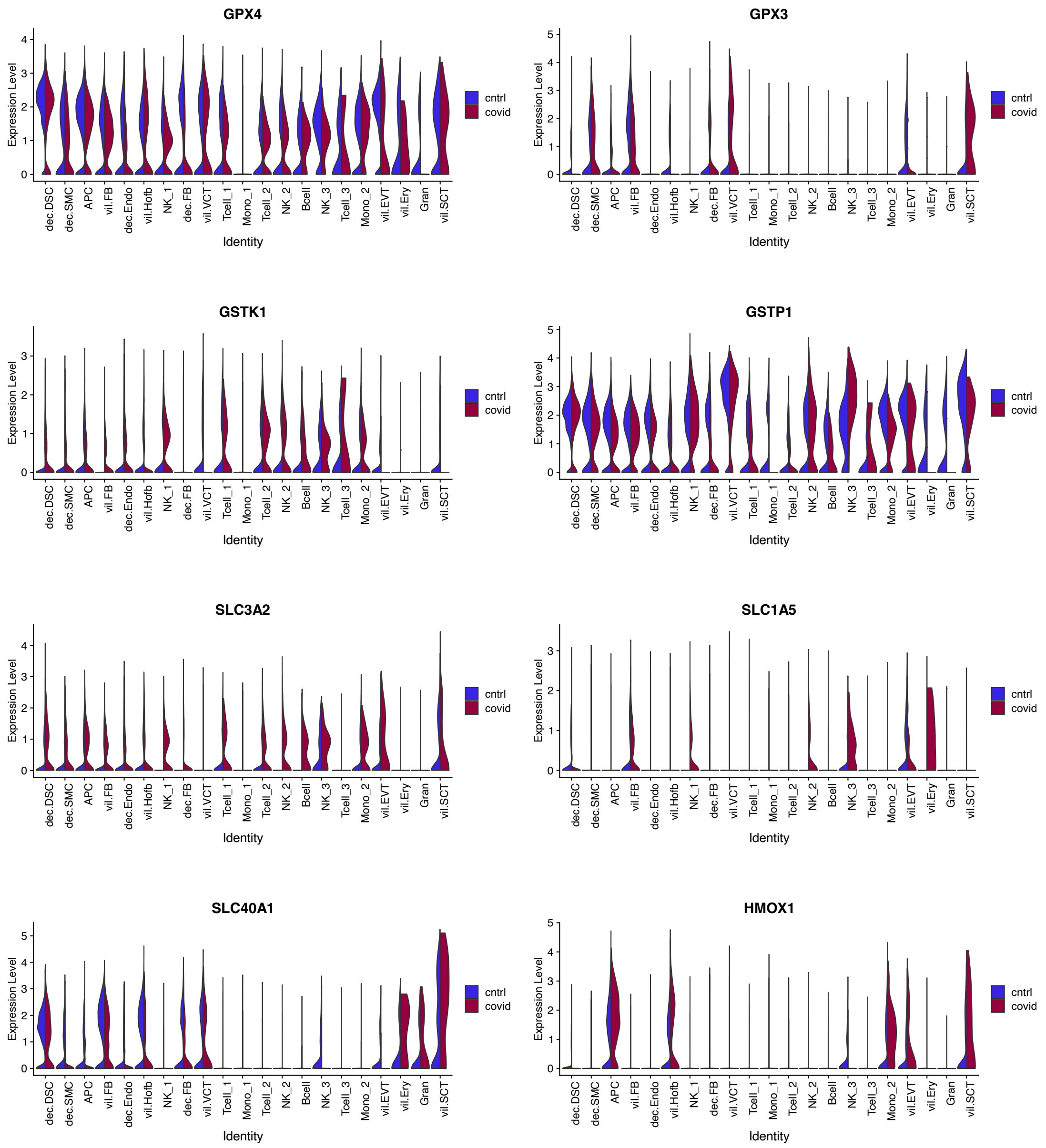

Supplementary Figure 3. Cell-type specific expression profiles of antioxidant and and iron/amino acid transport genes in placentas from SARS-CoV-2 infected and uninfected individuals. Violin plots display the mean single-cell RNA expression levels of genes involved in antioxidant defense and iron/amino acid transport across distinct placental cell types from SARS-CoV-2–positive (covid, red) and control (cntrl, blue) samples. Data are derived from the GSE171381 dataset and correspond to the cell clusters shown in Figure 6C. Genes shown include glutathione peroxidases (GPX4, GPX3), glutathione S-transferases (GSTK1, GSTP1), amino acid transporters (SLC3A2, SLC1A5), the iron exporter ferroportin (SLC40A1), and heme oxygenase 1 (HMOX1). Cell populations include dec.DSC (decidual stromal cell), dec.Endo (decidual endothelial cell), dec.SMC (decidual smooth muscle cell), dec.FB (decidual fibroblast), vil.FB (villous fibroblast), vil.EVT (villous extravillous trophoblast), vil.VCT (villous cytotrophoblast), vil.SCT (villous syncytiotrophoblast), vil.Ery (villous erythrocyte/blast), vil.Hofb (villous Hofbauer cell), APC (antigen-presenting cell), Mono_1 (monocyte subtype 1), Mono_2 (monocyte subtype 2), Gran (granulocyte), NK_1 (natural killer cell subtype 1), NK_2 (natural killer cell subtype 2), NK_3 (natural killer cell subtype 3), Tcell_1 (T cell subtype 1), Tcell_2 (T cell subtype 2), Tcell_3 (T cell subtype 3), Bcell (B cell).

Uncropped western blot images:

Figure 2

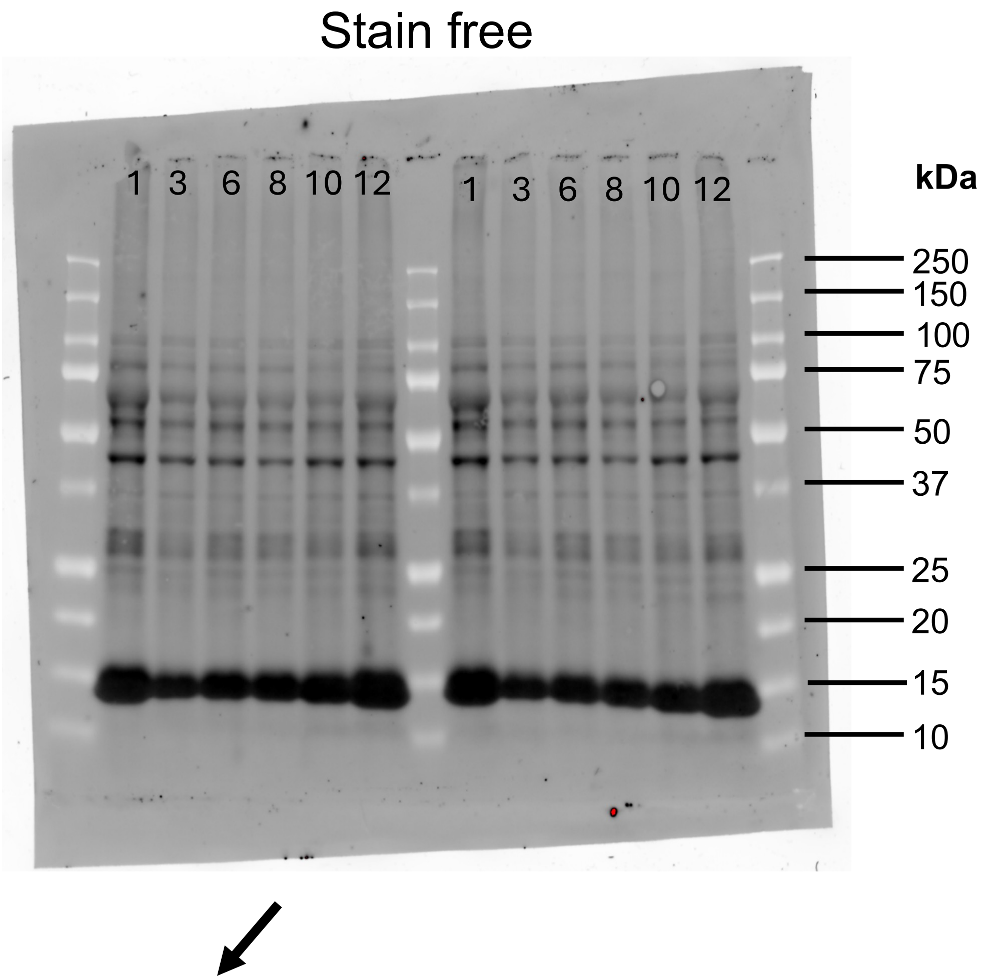

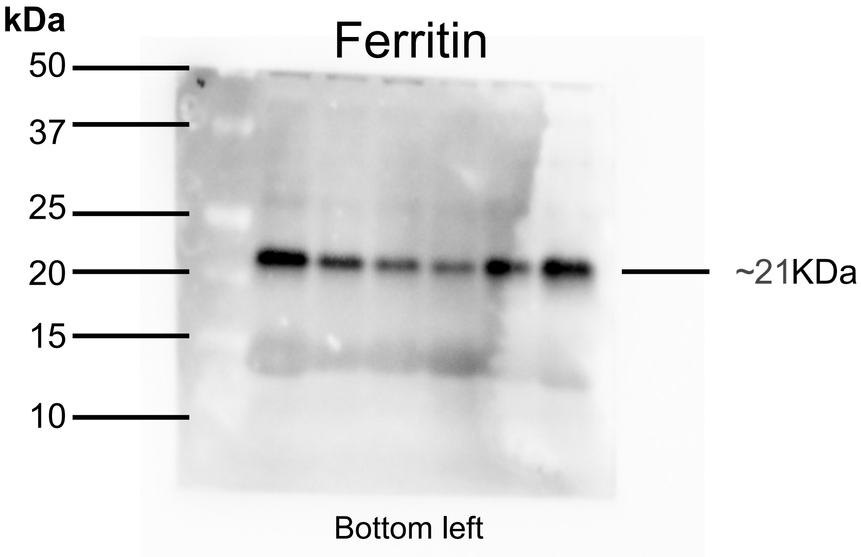

Figure 2

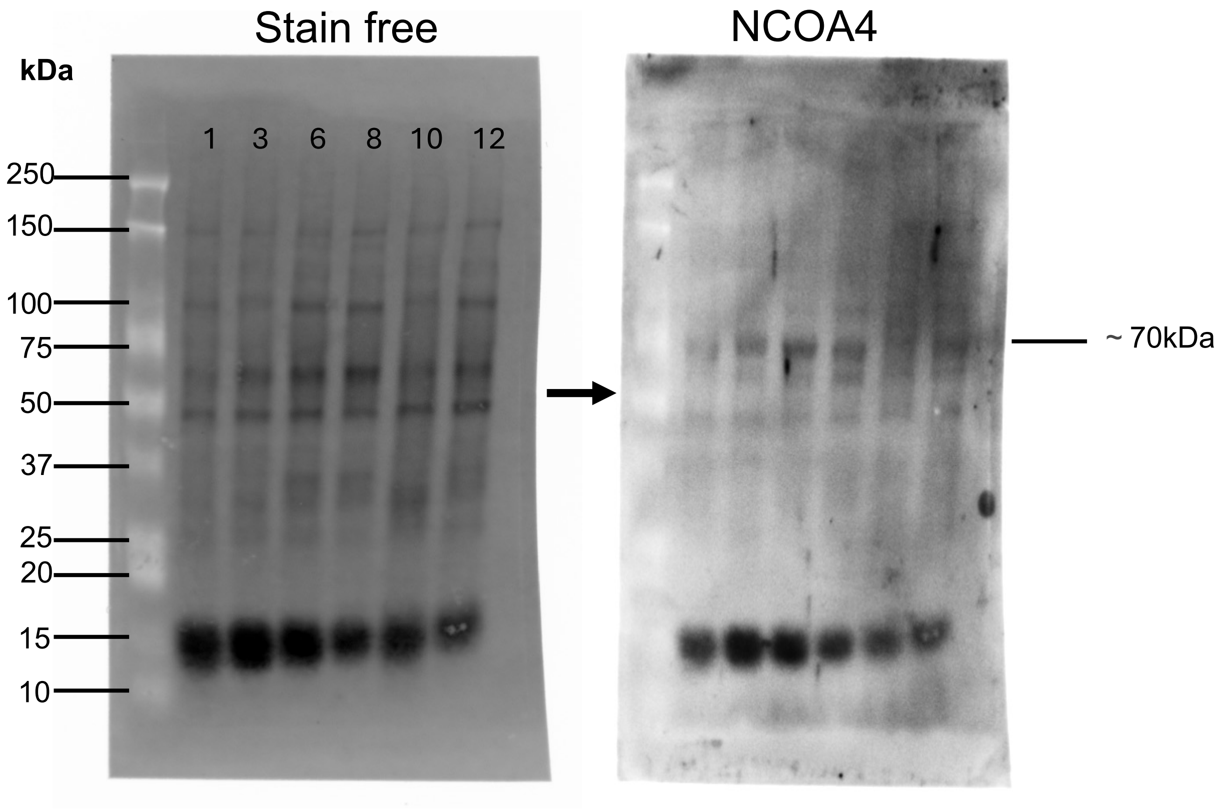

Stain free
NCOA4
kDa
1 3 6 8 10 12
250
150
100
75
50
37
25
20
15
10
~ 70kDa

Figure 3

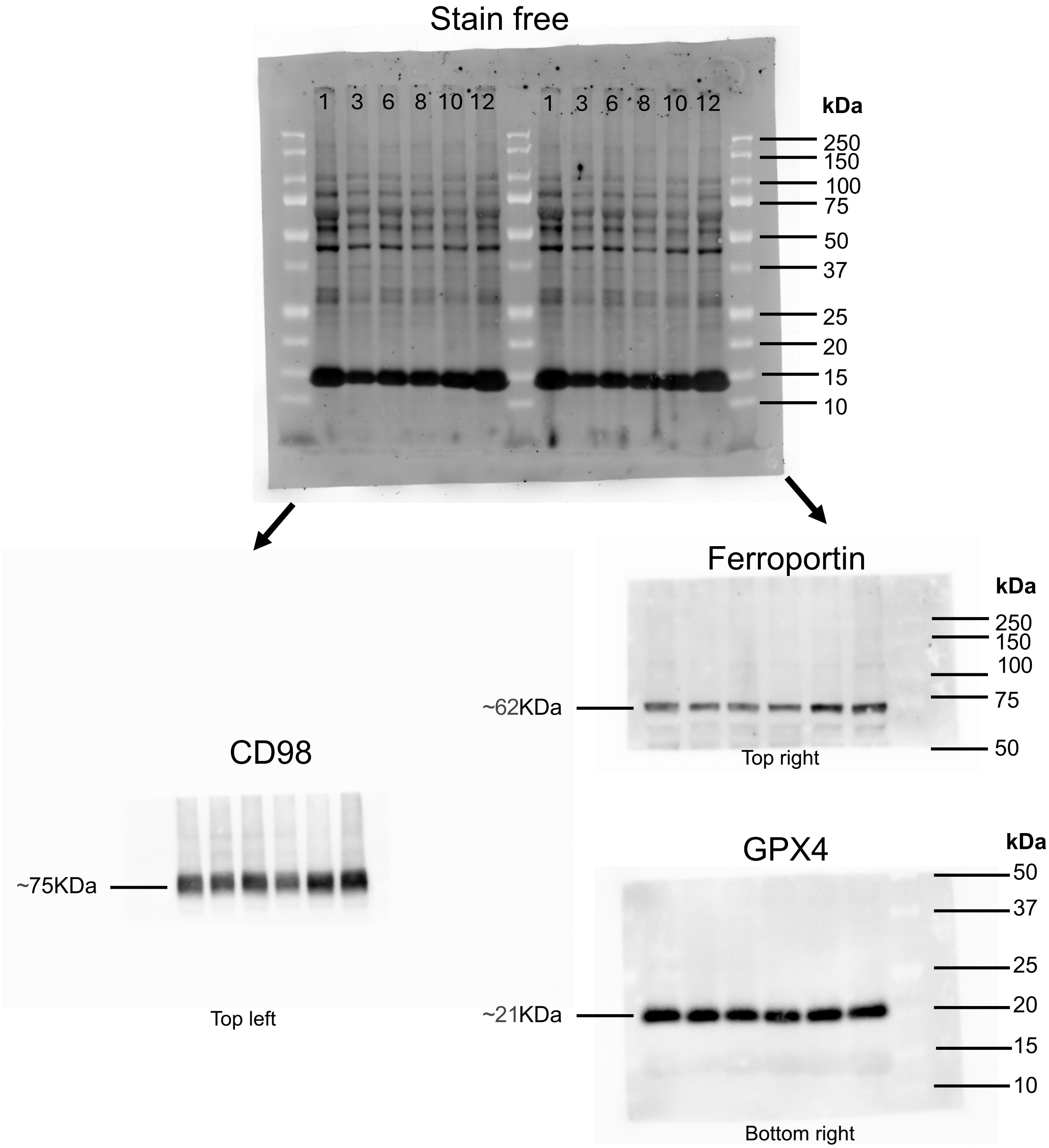
Stain free
1 3 6 8 10 12 1 3 6 8 10 12
kDa
250
150
100
75
50
37
25
20
15
10
Ferroportin
kDa
250
150
100
75
~62KDa
50
Top right
CD98
~75KDa
Top left
GPX4
kDa
50
37
25
~21KDa
20
15
10
Bottom right
